# Decrease in the production of beta-amyloid by berberine inhibition of the expression of beta-secretase in HEK293 cells

**DOI:** 10.1186/1471-2202-12-125

**Published:** 2011-12-12

**Authors:** Feiqi Zhu, Fujun Wu, Ying Ma, Guangjian Liu, Zhong Li, Yong'an Sun, Zhong Pei

**Affiliations:** 1 Neurology department of the affiliated Yuebei people's hospital, Shantou University Medical College. Shaoguan city, Guangdong Province, 512026, China; 2 Neurology department of Taihe hospital, the affiliated hospital of Hubei University of Medicine. Shiyan city, Hubei Province, 442000, China; 3 College of life Sciences Anhui Agricultural University, Hefei, Anhui, 230036, China; 4 Cardiology department of the affiliated Yuebei people's hospital, Shantou University Medical College. Shaoguan city, Guangdong Province, 512026, China; 5 Neurology department of the first affiliated hospital, Sun Yat-Sen University, Guangzhou city, Guangdong Province, 510080, China; 6 Neurology department of Peking University first hospital, Beijing, 100034, China

**Keywords:** Alzheimer's disease, berberine, beta-amyloid_40/42_, beta-secretase, extracellular signal-regulated kinase1/2

## Abstract

**Background:**

Berberine (BER), the major alkaloidal component of *Rhizoma coptidis*, has multiple pharmacological effects including inhibition of acetylcholinesterase, reduction of cholesterol and glucose levels, anti-inflammatory, neuroprotective and neurotrophic effects. It has also been demonstrated that BER can reduce the production of beta-amyloid_40/42_, which plays a critical and primary role in the pathogenesis of Alzheimer's disease. However, the mechanism by which it accomplishes this remains unclear.

**Results:**

Here, we report that BER could not only significantly decrease the production of beta-amyloid_40/42 _and the expression of beta-secretase (BACE), but was also able to activate the extracellular signal-regulated kinase1/2 (ERK1/2) pathway in a dose- and time-dependent manner in HEK293 cells stably transfected with APP695 containing the Swedish mutation. We also find that U0126, an antagonist of the ERK1/2 pathway, could abolish (1) the activation activity of BER on the ERK1/2 pathway and (2) the inhibition activity of BER on the production of beta-amyloid_40/42 _and the expression of BACE.

**Conclusion:**

Our data indicate that BER decreases the production of beta-amyloid_40/42 _by inhibiting the expression of BACE via activation of the ERK1/2 pathway.

## Background

Alzheimer's disease (AD) is the most prominent form of senile dementia. In the pathogenesis of AD, amyloid-β peptide (Aβ) plays a critical and primary role [1]. The aggregation and accumulation of extracellular and intracellular Aβ_40/42 _impairs synaptic plasticity and memory [2,3]. Aβ_40/42 _is generated by β-secreatase- (beta-site amyloid precursor protein cleaving enzyme, BACE) and γ-secretase-mediated sequential cleavages of amyloid precursor protein (APP). Inhibition of the production of Aβ_40/42 _can be expected to delay the development of AD [4]. In fact, some nonsteroidal anti-inflammatory drugs (NSAIDs), including sulindac sulfide, S-ibuprofen, R-ibuprofen and indomethacin, have been shown to inhibit the production of Aβ_40/42 _by inhibiting the expression of BACE and the activity of γ-secretase via activating peroxisome proliferator-activated receptor γ (PPAR γ) and inhibiting Rho-Rho associated kinase (Rho-ROCK) pathway [5,6]. Additionally, some statins, including sinvastatin, rosuvastatin, and lovastatin, the cholesterol-lowering drugs, have been found to reduce levels of Aβ_40/42 _by promoting the expression of α-secretase and inhibiting BACE activity [7-9].

Berberine (BER), an isoquinoline alkaloid existing in *Cortex phellodendri *(Huangbai) and *Rhizoma coptidis *(Huanglian), has a long history in China as a non-prescription drug for the treatment of diarrhea and gastrointestinal disorders. In recent years, many studies have indicated that BER has multiple pharmacological effects. BER is a novel cholesterol-lowering drug distinct from the statin family. It works by increasing the expression of low-density lipoprotein receptors (LDLR) and inhibiting lipid synthesis [10,11]. BER can also improve insulin resistance and exerts an insulin-independent glucose-lowering effect, stimulating insulin secretion and sensitizing insulin activity, inducing glycolysis, and increasing glucose transport and uptake activity [12-17]. At the same time, some studies have found that BER exerts anti-inflammatory effects by inhibiting arachidonic acid metabolism and the production of some inflammatory factors including cyclooxygenase-2 (COX-2), interleukin-1 beta (IL-1β), tumor necrosis factor-alpha (TNF-α), Interleukin-1 (IL-6) and inductible nitric oxide synthase (iNOS)[18-23].

BER can pass through the blood-brain barrier and reach the brain parenchyma in a dose- and time-dependent manner [24], and has multiple neuropharmacological properties including neuroprotective and neurotrophic effects. It also stimulates anti-neuronal apoptosis, improves cerebral microcirculation, reduces depression, and inhibits acetylcholinesterase [25-27]. Notably, one study [28] has reported that BER can decrease the production of Aβ_40/42_, but the mechanism remains unclear. Further investigation of how BER inhibits the expression of BACE may have significant impact on the treatment of AD. In this study, we therefore focused on the mechanism of BER on BACE and Aβ_40/42 _inhibition, using HEK293 cells stably transfected with APP695 containing the Swedish mutation.

## Results

### Effects of BER and U0126 on the proliferation and cytotoxicity of HEK293 cells

**Figure 1:**
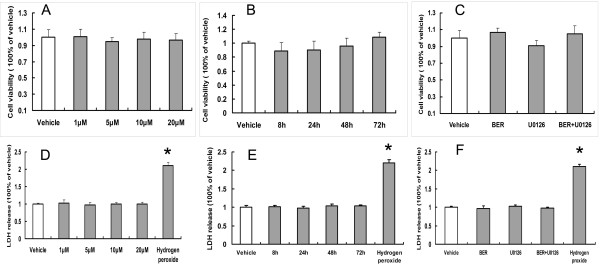
**Evaluation of the treatments on the proliferation and the cytotoxicity of HEK293 cells by MTT assay and LDH assay**. (A) Effects of BER (1 μM, 5 μM,10 μM, and 20 μM) on the proliferation of HEK293 for 48 hours of incubation, *P *> 0.05 compared with vehicle-treated group (n = 5). (B) Effects of BER (5 μM) on the proliferation of HEK293 for 8, 24, 48, and 72 hours of incubation, *P *> 0.05 compared with vehicle-treated group (n = 5). (C). Effects of BER (5 μM), U0126 (0.5 μM), and U0126 with BER (0.5 μM+5 μM) on the proliferation of HEK293 cells for 48 hours of incubation, *P *> 0.05 compared with vehicle-treated group (n = 5). (D) Effects of BER (1 μM, 5 μM, 10 μM, and 20 μM) on the cytotoxicity of HEK293 for 48 hours of incubation, *P < 0.05 when compared with vehicle-treated group (n = 5). (E) Effects of BER (5 μM) on the cytotoxicity of HEK293 for 8, 24, 48, and 72 hours of incubation, *P < 0.05 when compared with vehicle-treated group (n = 5). (F). Effects of BER (5 μM), U0126 (0.5 μM), and U0126 with BER (0.5 μM+5 μM) on the cytotoxicity of HEK293 cell for 48 hours of incubation, *P < 0.05 when compared with vehicle-treated group (n = 5).

The MTT assay was used to detect the treatments on the proliferation of HEK293 cells. Relative to the vehicle group, no significant declines were observed in the cells receiving treatments (*P *> 0.05) (Figure 1A, B and 1C). The LDH release of cultured medium was used to assay the treatments for the cytotoxicity of HEK293 cells. Compared with vehicle treatment, BER and U0126 showed no significant effects on the release of LDH in the culture medium (*P *> 0.05) (Figure 1D, E and 1F), but 3% H_2_O_2 _significantly increased the release of LDH in the culture medium (*P *< 0.01).

### Effects of BER and U0126 on the production of Aβ_40/42_

**Figure 2:**
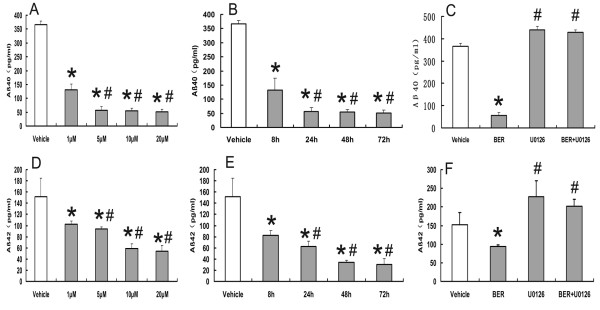
**Evaluation of the treatments on the production of Aβ40/42 in HEK293 cell by ELISA**. (A) BER (1 μM, 5 μM, 10 μM, and 20 μM) can inhibit the production of Aβ40 for 48 hours of incubation,*P < 0.01 compared with vehicle-treated group, #*P *< 0.05 compared with BER (1 μM) groups (n = 5). (B) BER (5 μM) can inhibit the production of Aβ40 from 8 to 72 hours of incubation, *P < 0.01 compared with vehicle-treated group, #*P *> 0.05 compared with BER (8 h) groups (n = 5). (C) U0126 (0.5 μM) can abolish the inhibition of BER (5 μM) on the production of Aβ40, *P < 0.01 compared with vehicle-treated group, #*P *< 0.05 compared with vehicle-treated group (n = 5). (D) BER (1 μM, 5 μM, 10 μM, and 20 μM) can inhibit the production of Aβ42 for 48 hours of incubation,*P < 0.01 compared with the vehicle-treated group, #*P *< 0.05 compared with BER (1 μM and 5 μM) groups (n = 5). (E) BER (5 μM) can inhibit the production of Aβ42 from 8 to 72 hours of incubation, *P < 0.01 compared with the vehicle-treated group, #*P *< 0.05 compared with BER (8- and 24-hour) groups (n = 5). (F) U0126 (0.5 μM) can abolish the inhibition of BER (5 μM) on the production of Aβ42, *P < 0.01 compared with vehicle-treated group, #*P *< 0.05 compared with vehicle-treated group (n = 5).

We assayed the treatments on the extracellular Aβ_40/42 _levels in the medium cultured from HEK293 cells by sandwich ELISA. BER (1 μM, 5 μM, 10 μM, and 20 μM) significantly reduced the levels of Aβ_40 _(366.2 ± 13.5 pg/ml vehicle control, 131.5 ± 20.8 pg/ml at 1 μM, 56.2 ± 13.5 pg/ml at 5 μM, 54.4 ± 9.2 pg/ml at 10 μM, and 50.8 ± 10.2 pg/ml at 20 μM) for 48 hours of incubation (Figure 2A) and the levels of Aβ_42 _(152.1 ± 32.9 pg/ml vehicle control, 102.3 ± 5.9 pg/ml at 1 μM, 94.3 ± 3.5 pg/ml at 5 μM, 58.4 ± 9.1 pg/ml at 10 μM, and 54.3 ± 10.1 pg/ml at 20 μM) for 48 hours of incubation (Figure 2D). BER (5 μM) significantly reduced the levels of Aβ_40 _from 8 hours to 72 hours of incubation (366.2 ± 13.5 pg/ml vehicle control, 131.5 ± 43.8 pg/ml at 8 hours, 58.3 ± 13.4 pg/ml at 24 hours, 56.2 ± 13.5 pg/ml at 48 hours, and 50.8 ± 10.2 pg/ml at 72 hours) (n = 5) (Figure 2B) and the levels of Aβ_42 _from 8 hours to 72 hours of incubation (152.1 ± 32.9 pg/ml vehicle control, 82.3 ± 9.1 pg/ml at 8 hours, 62.2 ± 10.5 pg/ml at 24 hours, 34.5 ± 3.5 pg/ml at 48 hours, and 30.5 ± 10.3 pg/ml at 72 hours) (Figure 2E). U0126 (0.5 μM) was found to significantly alleviate the reduction of BER (5 μM) on the production of Aβ_40/42_. The Aβ_40 _levels of the vehicle, BER (5 μM), U0126 (0.5 μM), and U0126 with BER groups (0.5 μM+5 μM) were 366.2 ± 13.5 pg/ml, 56.2 ± 13.5 pg/ml, 439.2 ± 5.6 pg/ml, and 429.2 ± 11.2 pg/ml, respectively (Figure 2C). The Aβ_42 _levels of the vehicle, BER (5 μM), U0126 (0.5 μM), and U0126 with BER (0.5 μM+5 μM) are 152.1 ± 32.9 pg/ml, 94.3 ± 3.5 pg/ml, 227.94 ± 41.9 pg/ml, and 202.06 ± 18.3 pg/ml, respectively (Figure 2F).

### Effects of BER and U0126 on the expression of BACE

**Figure 3:**
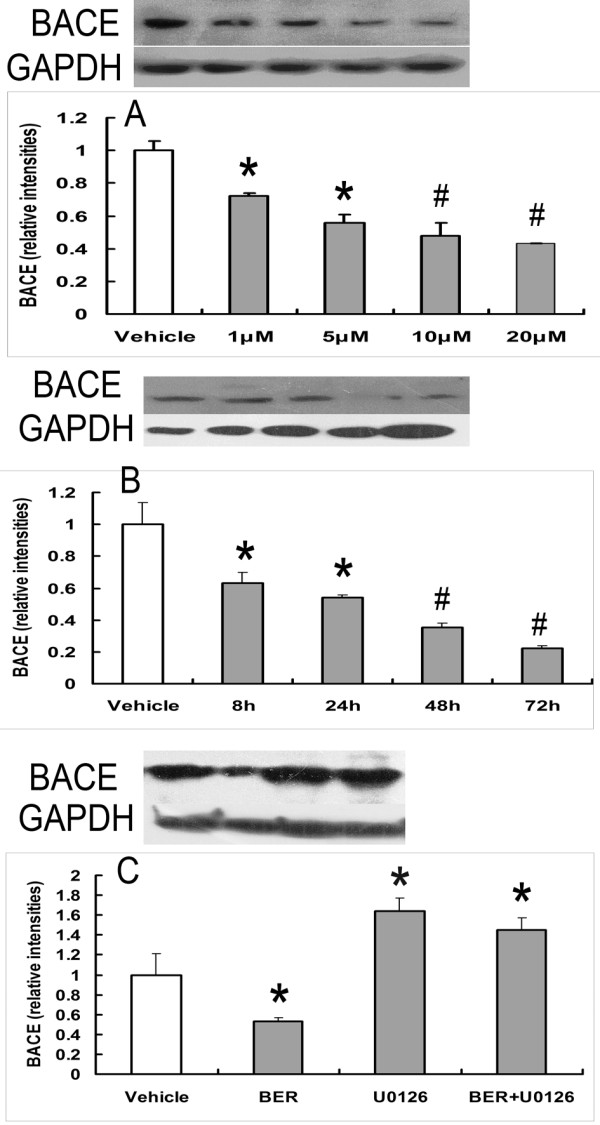
**Evaluation of the treatments on the expression of BACE by Western blot**. (A) BER (1 μM, 5 μM, 10 μM, and 20 μM) can significantly decrease the expression of BACE in a dose-dependent manner for 48 hours of incubation, *P < 0.05 compared with vehicle-treated group, #*P *< 0.01 compared with vehicle-treated group (n = 3). (B) BER (5 μM) can decrease the expression of BACE from 8 to 72 hours of incubation, *P < 0.05 compared with vehicle-treated group, #*P *< 0.01 compared with the vehicle-treated group (n = 3). (C) BER (5 μM) can decrease the expression of BACE for 48 hours of incubation, U0126 (0.5 μM), and BER with U0126 (5 μM +0.5 μM) can increase the expression of BACE for 48 hours of incubation, *P < 0.05 compared with vehicle-treated group (n = 3).

We assayed the expression of BACE in HEK293 cells by WB. BER (1 μM, 5 μM, 10 μM, and 20 μM) was found to significantly reduce the expression of BACE for 48 hours of incubation (Figure 3A). BER (5 μM) was found to significantly reduce the expression of BACE for 8 hours, 24 hours, 48 hours, and 72 hours of incubation (Figure 3B). However, U0126 (0.5 μM) was found to significantly increase the expression of BACE and alleviate the inhibition of BER (5 μM) on the expression of BACE (Figure 3C).

### Effects of BER and U0126 on the activation of ERK1/2 pathway

**Figure 4:**
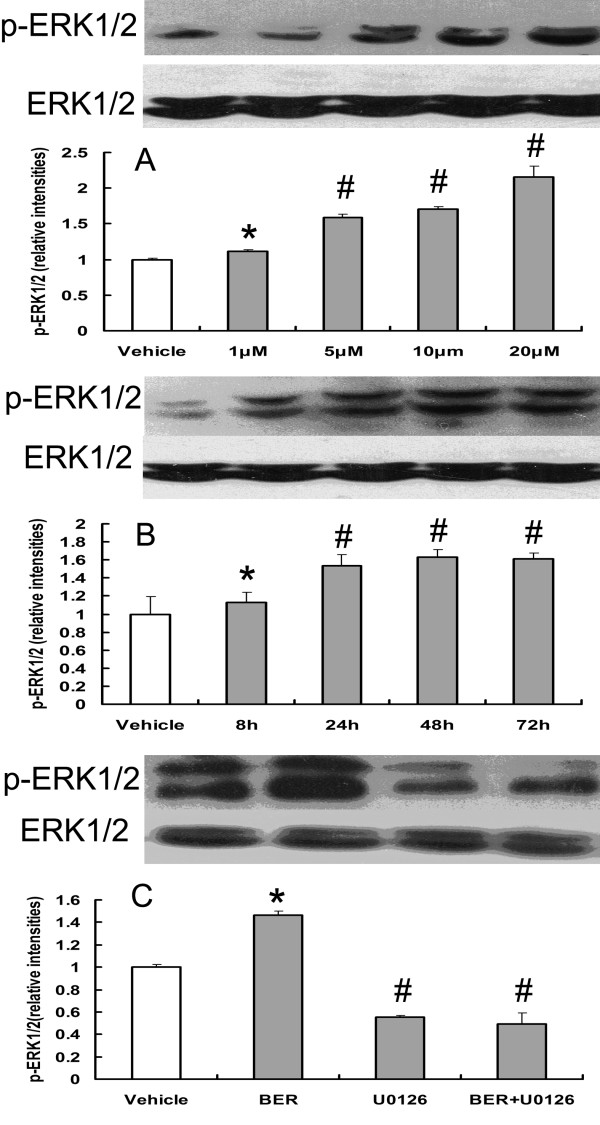
**Evaluation of the treatments on the expression of the activation of ERK1/2 pathway by Western blot**. BER (1 μM, 5 μM, 10 μM, and 20 μM) can significantly increase phosphorylation-ERK1/2 for 48 hours of incubation, *P < 0.05 compared with vehicle-treated group, #*P *< 0.01 compared with vehicle-treated group (n = 3). (B) BER (5 μM) can increase phosphorylation-ERK1/2 from 8 to 72 hours of incubation, *P < 0.05 compared with vehicle group, #*P *< 0.01 compared with vehicle-treated group (n = 3). (C) U0126 (0.5 μM) can completely abolish the activation of BER on ERK1/2, *P < 0.05 compared with the vehicle-treated group, #*P *< 0.01 compared with vehicle-treated group (n = 3).

We detected the effects of BER on the activation of ERK1/2 pathway by WB. We found that BER (1 μM, 5 μM, 10 μM, and 20 μM) significantly increased the expression level of p-ERK1/2 for 48 hours of incubation (Figure 4A). BER (5 μM) significantly increased the expression level of p-ERK1/2 for 8 hours, 24 hours, 48 hours, and 72 hours of incubation (Figure 4B). However, U0126 (0.5 μM) significantly inhibited the activation of ERK1/2 and abolished the activation effect of BER (5 μM) on ERK1/2 for 48 hours of incubation (Figure 4C).

## Discussion

In this study, we observed that BER significantly decreased the production of Aβ_40/42 _and the expression of BACE via activation of the ERK1/2 pathway in a dose- and time-dependent manner. We also found that U0126, an antagonist of ERK1/2 pathway, abolished the effects of BER on both Aβ_40/42 _and BACE. BER had previously been demonstrated to be able to reduce cancerous conditions by inhibiting the proliferation of tumor cells [29,30], but we did not find that BER could inhibit the proliferation and show cytotoxicity toward HEK 293 cells by MTT and LDH assays. From this, it can be concluded that the inhibition of BER on the production of Aβ_40/42 _is not associated with the anti-proliferative or cytotoxic qualities of BER.

The enzyme BACE is crucial to the production of Aβ_40/42 _and the expression of BACE increases in the brains of AD patients [5]. For this reason, BACE has been considered as a therapeutic target for AD treatments. On the other hand, the expression and activity of BACE is regulated by the ERK1/2 pathway in a dose- and time-dependent manner [31], and BER increases the expression of LDLR and glucose uptake by activating the ERK1/2 pathway [10,15]. So berberine-induced reduction of BACE1 protein levels is related to ERK1 activation. Furthermore, though BER has been shown unable to inhibit the activity of BACE *in vitro *[25], the ERK1/2 pathway negatively modulates BACE1 activity *in vivo *[31]. Thus, we think that BER might also decrease the production of Aβ_40/42 _by inhibit BACE1 activity via activating ERK1/2 pathway, and it need to be studied in the next study.

At the same time, BER may decrease the production of Aβ_40/42 _by affecting the activity of α-secretase and γ-secretase. It has been reported that ERK1/2 is an endogenous negative regulator of γ-secretase activity, and NSAIDs can inhibit γ-secretase activity by inhibiting the Rho-ROCK pathway [6,32,33]. BER inhibits tumor cell migration by inhibiting the Rho-ROCK pathway in HONE1 cells [34], so it is possible that BER inhibits the activity of γ-secretase by activating the ERK1/2 pathway and inhibiting the Rho-ROCK pathway. Moreover, BER, an acetylcholinesterase inhibitor, may be able to upregulate α-secretase activity by promoting the translocation of α-secretase to the cell surface [35]. All these possibilities require further study.

## Conclusion

In this study, we demonstrated that BER can decrease the production of Aβ_40/42 _by inhibiting the expression of BACE via activation of the ERK1/2 pathway. In previous studies, we demonstrated that BER improved impaired spatial memory and increased both the activation of microglia and the expression of insulin degrading enzyme (IDE) in the rat model of AD [36-38]. Other researchers have demonstrated other pharmacological effects of BER in HEK293 cells, e.g., inhibiting Aβ_42 _aggregation and attenuating the Tau hyperphosphorylation induced by calyculin A [39,40]. Together, we consider BER to be a very promising drug for use in AD patients.

## Methods

### Cell culture and treatments

HEK293 cells stably transfected with APP695 containing the Swedish mutation were maintained in Dulbecco's modified Eagle's medium (DMEM), supplemented with 5% fetal bovine serum and G418 (Sigma, St. Louis, MO, U.S.) (100 μg/mL) in a humidified atmosphere at 37°C with 5% CO2. HEK293 cells were given BER (Sigma, St. Louis, MO, U.S.) (1 μM, 5 μM, 10 μM, and 20 μM), U0126 (Sigma, St. Louis, MO, U.S.) (0.5 μM), and BER with U0126 (5 μM+0.5 μM) for 48 hours, and HEK293 cells were also given BER (5 μM) for 8 hours, 24 hours, 48 hours and 72 hours.

### MTT analysis

After the cells were treated in the manner described above, 10 μl of 1 mg/ml MTT stock (Sigma, St. Louis, MO, U.S.) were added to each well and the incubation continued for another 4 hours. One hundred microliters of a solution containing 20% SDS and 50% dimethylformamide (pH 4.8) were then added to each well. After overnight incubation, absorption values at a wavelength of 570 nm were determined by spectrophotometer.

### Cellular toxicity analysis

HEK293 cells were plated at a density of approximately 1 × 10^4 ^cells per well on 24-well plates. After 24 hours of incubation, the conditioned media were replaced with new media containing BER, U0126, and BER with U0126 at the final concentrations and the final times indicated. Lactate dehydrogenase (LDH) activity was determined to evaluate the cell toxicity of BER, U0126, and BER with U0126 by using cytotoxicity detection kits (Njjcbio Institute, China) according to the manufacturer's instructions. Hydrogen peroxide (3%) was used as a positive control and added to the conditioned media during the last hour of incubation. The baseline was determined in control wells containing no cells and the values obtained there were subtracted from those obtained from experimental wells.

### Sandwich ELISA

HEK293 cells were plated at a density of approximately 4 × 10^4 ^cells per well on 6-well plates. After 24 hours of incubation, the conditioned media were replaced by new media containing BER, U0126, and BER with U0126 at the final concentrations and final times indicated. The cultured media were harvested and extra cellular Aβ levels were determined by using the Human Aβ_40/42 _Assay Kit (Cusabiao Biotech Co., Ltd., U.S.) according to the manufacturer's instructions.

### Western blotting (WB) analysis

HEK293 cells were plated at a density of 4 × 10^4 ^cells per well on 6-well plates. After 24 hours of incubation, the conditioned media were replaced by new media containing BER, U0126, and BER with U0126 at the final concentrations and final times indicated. Cells were lysed in a cell and tissue protein extraction reagent and protease inhibitor cocktail and phosphotase inhibitor cocktail (Kangchen Bio-tech, China), phenyl-methyl-sulfonyl-fluoride-proteomics grade kit (Kangchen Bio-tech, China). Protein extracts (protein 50 μg) were subjected to SDS-PAGE. The levels of BACE, p-ERK1/2, ERK1/2, and GAPDH in the cell lysates were quantified by WB analysis using polyclonal antibody anti-BACE (487-501, 1:10000 dilution, EMD Bioscience, Germany), monoclonal antibody anti-phospho-ERK1/2 and ERK1/2 (137F5 and 197G2, 1:1000 dilution, Cell Signaling Technology, U.S.), and polyclonal antibody anti-GAPDH (IBN9003L, 1:3000 dilution, KB Biotech, China), respectively. This was followed by application of peroxidase-conjugated secondary antibodies (GGHL-15P,1:5000 dilution, ICL Lab, U.S.). Immunoreactive signals were detected by enhanced chemiluminescence using ECL Plus WB detection reagents (Pierce); signal intensity was determined with a densitometer, LAS-3000 (Fuji Photo Film Co., Ltd., Tokyo, Japan). The amounts of immunoreactive BACE on internal control GAPDH and p-ERK1/2 on internal control ERK1/2 in each sample were calculated by using Quality One software (Bio-Red, U.S.).

### Statistical analysis

All of the data were expressed as mean ± SD and the analysis was carried out using the one way analysis of variance (ANOVA). Values of *P *< 0.05 were considered statistically significant.

## Abbreviations

Aβ: beta-amyloid; AD: Alzheimer's disease; BACE: beta-secretase; BER: berberine; ELISA: enzyme-linked immunosorbent assay; ERK1/2: extracellular signal-regulated kinase1/2; LDH: lactate dehydrogenase; WB: western blot.

## Competing interests

All authors disclose the following:

(a) There are no actual or potential conflicts of interest, including any financial, personal, or other relationships with other people or organizations within 3 years of the beginning of the work submitted that could inappropriately influence (bias) this work.

(b) This study does not contain data from human or animal subjects.

## Authors' contributions

All authors contribute equally to the study. All authors read and approved the final manuscript.
